# A Case of a Pressure Injury Where Electrical Stimulation Led to Team Cohesion and Enhanced Healing

**DOI:** 10.7759/cureus.82842

**Published:** 2025-04-23

**Authors:** Miki Tanaka, Hiroyuki Okuno, Akiyo Asai, Yoshiyuki Yoshikawa

**Affiliations:** 1 Department of Rehabilitation, Setsunan General Hospital, Kadoma, JPN; 2 Department of Rehabilitation, Nara Higashi Hospital, Tenri, JPN; 3 Department of Nursing, Setsunan General Hospital, Kadoma, JPN; 4 Graduate School of Rehabilitation Sciences, Naragakuen University, Nara, JPN

**Keywords:** electrical stimulation, interprofessional collaboration, microcurrent, pressure injury, wound healing

## Abstract

Pressure injuries are grave sequelae of prolonged immobility, with each episode incurring an estimated US $30,000-70,000 in direct medical costs and often necessitating protracted management. Beyond the economic burden, these ulcers disrupt the skin’s barrier, heightening infection risk and predisposing patients to sepsis. Although electrical stimulation (ES) is endorsed by clinical guidelines, systematic reviews, and meta-analyses, rehabilitation professionals remain under-represented in pressure-injury care. We report the case of a woman in her 50s with quadriplegia who developed a stage IV pressure injury that failed to improve with standard wound care. Following a physical therapist’s recommendation, ES was initiated, which not only accelerated wound closure but also fostered interprofessional collaboration. Stage III/IV ulcers typically require approximately 150-240 days to heal; in contrast, this lesion closed in just 90 days*.* Rigorous, twice-daily wound assessment, pH-balanced cleansing, and scheduled repositioning were integral to this expedited outcome. The case underscores the value of team-based management and highlights ES as a promising adjunct for severe pressure injuries. Larger cohort studies are warranted to confirm these findings and refine clinical protocols.

## Introduction

Pressure injuries are serious complications that often occur in patients with prolonged bed rest or limited mobility [1]. Recent economic analyses estimate that managing a stage III/IV pressure injury typically requires six to eight months of active treatment [2,3] and incurs total costs of roughly US $30,000-70,000 per case, depending on wound severity and care setting [4,5]. In addition, pressure injuries weaken the skin’s barrier function, raising the risk of infection and possibly leading to sepsis [6]. Consequently, healthcare professionals must focus on both prevention and prompt treatment.

Effective management involves collaboration across multiple disciplines. Guidelines recommend positioning, skin care, patient education, exercise, and physical therapy to address pressure injuries following onset [7].

Electrical stimulation (ES) is supported by multiple systematic reviews and meta-analyses that demonstrate faster wound closure in pressure injuries [8-10], and this evidence has been incorporated into major clinical guidelines [7]. The 2019 International Guideline European Pressure Ulcer Advisory Panel, National Pressure Injury Advisory Panel, Pan Pacific Pressure Injury Alliance (EPUAP/NPIAP/PPPIA) assigns a grade A recommendation for pulsed-current ES in recalcitrant stage II-IV wounds [7], while the 2023 Wound Healing Society guideline issues a level I endorsement [11].

Its mechanisms include improving tissue perfusion [12], enhancing cell migration [13-15], promoting cell proliferation [16], inducing myofibroblast differentiation [17], and offering anti-inflammatory benefits [18]. Nevertheless, real-world adoption is still limited: recent surveys indicate that only about 10-15% of specialized wound-care clinics routinely employ ES for selected pressure-injury stages [19], even though observational data from those centers show shorter healing times compared with standard care alone [20]. Despite these benefits, rehabilitation professionals frequently have limited involvement in pressure-injury care. Contributing factors include documented knowledge gaps in wound management curricula, unclear role demarcation vis-à-vis nursing and surgical teams, heavy caseloads that constrain the time available for labor-intensive ulcer care, and the absence of dedicated reimbursement codes for certain adjunctive therapies such as electrical stimulation [19,21]. In the case described here, ES was proposed by a physical therapist and carried out in coordination with other healthcare providers, leading to both wound improvement and enhanced team cohesion.

## Case presentation

A woman in her 50s presented with fever and neck stiffness, resulting in a diagnosis of meningitis. During treatment, she developed quadriplegia and became almost entirely dependent in activities of daily living (ADL). On hospital day 14, prolonged immobility led to a pressure injury on her left gluteal region, classified as National Pressure Ulcer Advisory Panel (NPUAP) stage II with a DESIGN-R® score of 18-Depth 2, Exudate 3, Size 6, Infection/Inflammation 3, Granulation 3, Necrotic tissue 0, Pocket (Undermining) 0 (D2/E3 s6 I3 g3 N0 P0) where the composite score (range 0-66) reflects moderate severity [22-24]. By hospital day 21, the lesion had advanced to stage IV depth with the formation of an undermining pocket, yielding a DESIGN-R® score of 45 (D4/E6 s6 I3 g3 N3 P24). Undermining area was defined as tissue loss extending underneath intact skin, forming a subcutaneous pocket beyond the visible wound edges. This was measured by gently probing the wound perimeter with a sterile applicator. Relevant comorbidities included hypertension (average ward readings 128/78 mmHg while receiving amlodipine 5 mg/day) and type 2 diabetes mellitus with an HbA1c of 7.4%, indicating moderate glycaemic control; her Braden Scale score was 12. The DESIGN-R® score, devised by the Japanese Society of Pressure Ulcers, is used to assess and monitor pressure injury severity [22-24]. No severe infection necessitating systemic antibiotics occurred. Therefore, standard wound management (wound bed preparation) continued, supported by an air mattress and scheduled repositioning. At 28 days following the onset of the pressure injury, necrotic tissue was removed through gentle mechanical debridement during nurse-performed wound cleansing. This procedure was supplemented by the application of a topical povidone-iodine sugar ointment (Isodine Sugar), functioning as a chemical and enzymatic debriding agent. As a result, the DESIGN-R® score improved to 37 (D3/E6 s3 I3 g1 N0 P24). At this stage, a physical therapist recommended the initiation of ES, and its use was subsequently approved by the attending physician.

Electrical stimulation

**Figure 1:**
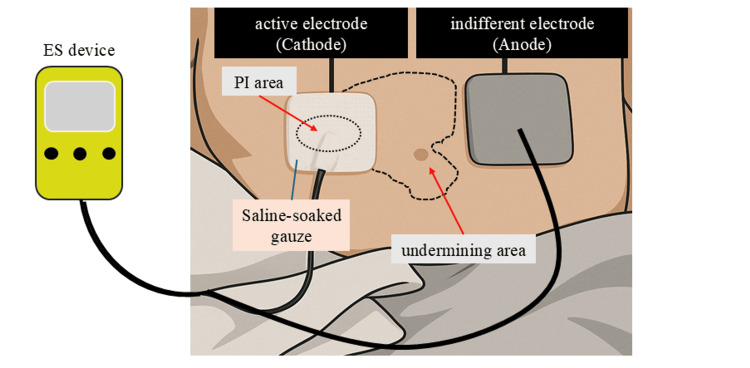
Schematic representation of electrical stimulation therapy. The active electrode (cathode) was placed directly over the pressure injury, while the indifferent electrode (anode) was positioned on the surrounding intact skin. The active electrode was wrapped in gauze moistened with normal saline to ensure adequate conductivity and was applied to the wound site. PI: pressure injury; ES: electrical stimulation. This illustration was created by first converting a clinical photograph of a pressure injury into an AI-generated image using an image-generation tool (ChatGPT).

An electrical stimulation device (iPES; Ito, Kawaguchi, Japan) was used to deliver the therapy. Monophasic pulsed microcurrent (MPMC) was selected because currents in the 180-220 µA range at 2 Hz have shown the greatest effect size for promoting wound contraction while causing minimal sensory perception [9,10,25,26]. MPMC is also relatively inexpensive and can be administered at the bedside, making it a practical option for patients with high care needs. Other options, such as vacuum-assisted closure (VAC) therapy or surgical reconstruction, were deferred because of the patient’s unstable clinical status and multiple comorbidities (Figure 1).

Following established protocols [26], we applied 200 µA at 2 Hz with a 250 ms pulse width for 60 minutes, five times weekly. The active electrode was placed over the wound and undermining area, and the indifferent electrode was placed on the contralateral side. Throughout each 60-minute session, the patient reported 0-1/10 pain, and no paraesthesia or erythema was observed, confirming good tolerability. Electrical stimulation (ES) was administered immediately after wound cleansing; nurses and physical therapists set up the equipment, removed it afterward, and resumed standard wound care.

Clinical course

**Figure 2:**
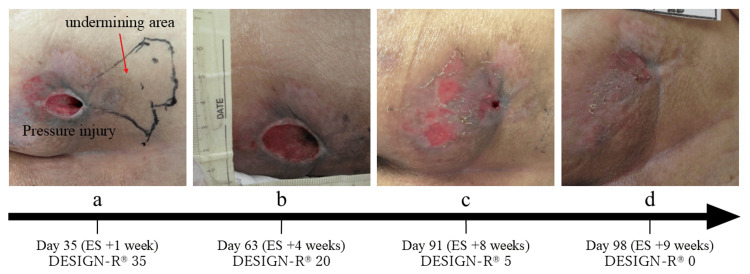
Progress of the pressure injury. (a) Day 35 (ES +1 week)–at the initiation of electrical stimulation (ES), the wound measured approximately 6.0 cm², with an undermining area of about 42.0 cm². (b) Day 63 (ES +4 weeks)–after three weeks of ES, the wound area had reduced to approximately 4.0 cm², and the undermining area had decreased to 20.0 cm². (c) Day 91 (ES +8 weeks)–at seven weeks post-ES initiation, the undermining had completely resolved. (d) Day 98 (ES +9 weeks)–by nine weeks, the wound had fully healed.

**Figure 3:**
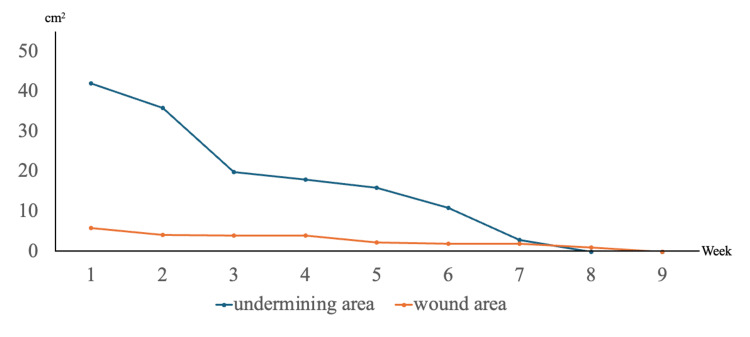
Changes in the wound area and undermining area. Starting from the initiation of electrical stimulation therapy, the undermining area began to decrease and had completely disappeared by seven weeks. Subsequently, the wound itself reduced in size and achieved complete healing at nine weeks.

One week after initiating ES, the wound area was about 6.0 cm², the undermining area was approximately 42.0 cm², and the DESIGN-R® score was 35 (D3/E6 s3 I1 g1 N0 P24) (Figure 2a). The wound area, undermining area, and DESIGN-R® score were assessed weekly. Four weeks after starting ES, the wound area had decreased to around 4.0 cm², the undermining area to 20.0 cm², and the DESIGN-R® score to 20 (D3/E3 s3 I1 g1 N0 P12) (Figure 2b). By the eighth week, the undermining had resolved, and the DESIGN-R® score was 5 (D3/E1 s3 I0 g1 N0 P0) (Figure 2c). The wound was fully closed by the ninth week, marking complete healing (Figure 2d). Figure 3 shows the changes in the wound and undermining areas.

Ethical considerations

Before preparing this report, both verbal and written explanations were given to the patient and family, and informed consent was obtained for using clinical data. Steps were taken to maintain confidentiality and privacy.

## Discussion

In this case, the introduction of ES appeared to accelerate the resolution of a stage IV pressure injury. According to Sanada et al. [23], a DESIGN-R® score of 18 or lower typically predicts healing within 30-90 days. Large cohort studies of standard care report median healing times of 150-240 days for stage III/IV pressure injuries [2,3]. In contrast, the present wound closed in 90 days, representing a 40-60% reduction in healing duration. This difference suggests that the combination of structured wound assessment, cleansing, repositioning, and monophasic pulsed microcurrent may have contributed meaningfully to the accelerated outcome. While a Cochrane Review indicates that electrical stimulation can aid wound contraction and healing [27], it remains unclear whether it uniformly shortens healing times. In this instance, however, ES not only facilitated wound contraction but also contributed to an overall reduction in healing duration. A study by Yoshikawa et al. [28] observed complete healing in seven cases treated with MPMC, suggesting beneficial impacts on wound contraction and healing speed.

Proposed mechanisms of MPMC-induced wound healing include enhanced cell migration [13-15], proliferation [16], differentiation into myofibroblasts [17], and anti-inflammatory activity [18]. Although the patient’s blood pressure was well controlled, her HbA1c of 7.4% lies within a range associated with diminished neutrophil function and reduced collagen deposition [29], factors that could have prolonged healing; yet the ulcer still resolved in 90 days, indicating that timely ES and rigorous local care may have offset the adverse influence of moderate hyperglycemia. The deployment of ES also appeared to strengthen interprofessional collaboration, evidenced by coordinated cleansing and repositioning schedules and joint wound evaluations by nurses and physical therapists. Each discipline also shared weekly wound scores and collaboratively evaluated the healing process, fostering a shared understanding of treatment progress that likely promoted faster recovery. Prior surveys show that rehabilitation professionals are often under-involved in pressure-injury management [21], but their active participation here likely contributed to the favorable outcome [30]. Regular cleansing is especially beneficial, as bacterial load can revert to pre-cleansing levels within 24 hours [31]. Accordingly, we instituted a standardized protocol of twice-daily irrigation with a pH-balanced solution, and we consider this scheduled cleansing to have been a further contributor to the accelerated healing observed. Hence, consistent wound assessment and care optimized the healing environment and enhanced the efficacy of standard interventions. Guidelines likewise emphasize the value of a team-based approach to pressure-injury care [7], underscoring the impact of coordinated multidisciplinary efforts.

Clinical implications and recommendations for practice

*Patient*-​​​​​*Selection Criteria*

ES may be considered for stage III/IV ulcers that have failed to progress after ≥30 days of optimal standard care, provided the patient (i) retains intact pain sensation, (ii) has no pacemaker or other implanted device within the electrode field, and (iii) is in stable cardiopulmonary condition.

Implementation Considerations

Begin ES once bioburden is controlled, employ single-patient lead sets to avoid cross-contamination, and re-evaluate weekly; discontinue therapy if the wound area does not decrease by ≥20% within four weeks. These practical steps can help clinicians integrate ES safely and effectively into routine pressure-injury management.

## Conclusions

Electrical stimulation effectively expedited the healing of a severe pressure injury and reinforced interprofessional collaboration. This case illustrates the positive influence that rehab staff involvement can exert on both wound closure and overall team dynamics. Larger-scale studies could further explore ES-related benefits and underlying mechanisms in advanced wound care.
